# After the Research Is Done: Legal Obligations for Participants in Household Exposure Studies

**DOI:** 10.1289/ehp.124-A206

**Published:** 2016-11-01

**Authors:** Julia R. Barrett

**Affiliations:** Julia R. Barrett, MS, ELS, a Madison, WI–based science writer and editor, is a member of the National Association of Science Writers and the Board of Editors in the Life Sciences.

Household chemical exposure studies are essential for assessing human health risks associated with indoor environments.^1^ But as in all studies involving human subjects, researchers must strive to ensure that individuals understand the risks incurred by participating in a study.^2^ ^,^ ^3^ For household exposure studies, these risks may include legal obligations placed upon participants once they learn of contamination issues in their homes. A new review provides an overview of these potential obligations along with guidance for sharing this information with participants.^1^

In the past, research participants rarely had the option of having their individual results reported back to them unless those results were clinically significant.^2^ ^,^ ^4^ This approach was justified on the presumption that any benefits to the participants could be outweighed by having to grapple with baseless fears or worrisome questions with no answers.^3^ ^,^ ^4^ However, in the last 15–20 years, study participants have increasingly been viewed as partners in research, and ethicists and researchers have encouraged reporting individual information back to participants.^2^ ^,^ ^3^

**Figure:**
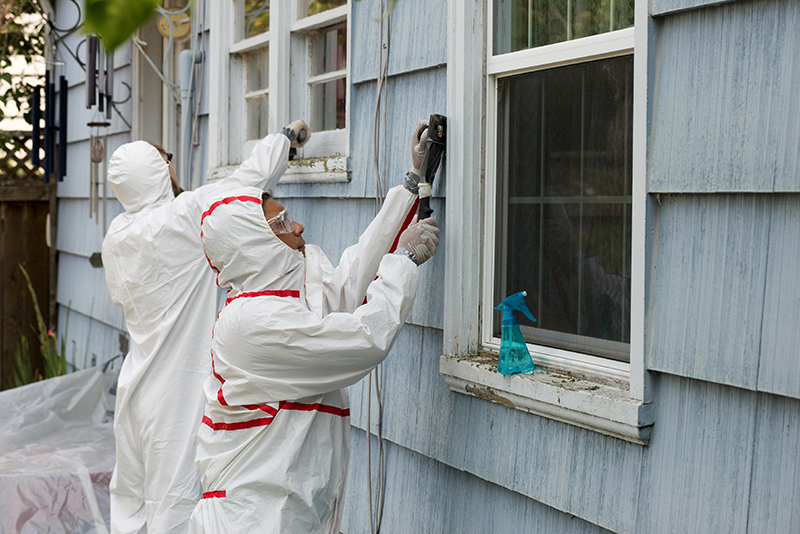
Some states have laws requiring landlords to remove or cover lead paint in homes occupied by young children. © Jamie Hooper/Alamy Stock Photo

Report back is not a one-size-fits-all endeavor, though, and researchers are ethically obligated to consider all aspects of how study results could affect individuals.^2^ ^,^ ^4^ Sharing that information is part of how researchers ensure that individuals can make an informed decision about whether to accept the risks and benefits of participating in a study.^2^

In the context of household exposure studies, report back reveals information such as the types and amounts of contaminants detected in participants’ homes.^2^ One risk of learning such results is that participants may then have a legal duty to clean up contamination or notify somebody else—such as a government regulator, tenant, or homebuyer—of the results. This possibility has not been assessed in depth, but both researchers and research participants have raised the issue.^1^ ^,^ ^5^ ^,^ ^6^

In the current review, Shaun Goho, a senior clinical instructor and staff attorney with the Emmett Environmental Law and Policy Clinic at Harvard Law School, delves into how report back could impose obligations on research participants under environmental, property transfer, and other laws. “The concern going into this research was that this could be an argument against report back. It could end up being a greater cost than a benefit to the participant,” he says.

Goho began by identifying relevant state and federal regulations, such as hazardous waste laws, real estate transfer and property rental laws, and tort laws. The laws fell into three categories. The first category included laws that impose clear legal duties on individuals, such as disclosing the presence of lead paint to prospective homebuyers. The second included laws from which research participants are clearly exempt, such as reporting requirements that involve much higher levels of hazardous emissions than are typically found in homes. The third included laws with ambiguous consequences as far as study participants go, such as requirements that tenants keep their rented home in a “safe and sanitary” condition.

He illustrated the legal implications for study participants in case studies where homes were found to contain lead, polychlorinated biphenyls (PCBs), polybrominated diphenyl ethers (PBDEs), or certain phthalates. Both lead and PCBs are well-known, stringently regulated human health hazards, and if levels were high enough, study participants would have to disclose or remediate the contamination. PBDEs (flame retardant components) and phthalates (plasticizers used in some building materials) are not as widely or as stringently regulated, and human health data are still emerging. Therefore, the presence of PBDEs or phthalates in a residents’ indoor air or dust is unlikely to carry any legal obligations. However, that situation could change in the future if regulatory limits are placed on these substances.^1^

Overall, Goho concludes, report back presents only limited, albeit real, legal risks to study participants, and these risks are not a compelling reason to withhold results. “Most of the time the potential legal risks are small enough, and when they do arise, they are outweighed by the benefits of receiving the information [about the presence of indoor pollutants],” he says.

Mónica Ramírez-Andreotta, an assistant professor in soil, water, and environmental science at the University of Arizona, agrees with this conclusion. “The majority of participants want to know about their environmental quality and want to take the steps to mitigate risk and protect their families,” she says. “In some cases, participants also want to know what they have to do with the data. For example, homeowners have asked whether they need to disclose their household data … when selling their homes.”

Ramírez-Andreotta, who was not involved in the study, emphasizes the broader importance of the guidance provided by the study. “I wouldn’t want to see any legal or liability discussion inhibit people from learning more about their environmental health and increasing their literacy regarding these issues,” she says. “As a community-engaged researcher, the more I can inform study participants and collaborators, the better.”
